# Lessons From Neuro-(a)-Typical Brains: Universal Multilingualism, Code-Mixing, Recombination, and Executive Functions

**DOI:** 10.3389/fpsyg.2020.00488

**Published:** 2020-04-23

**Authors:** Enoch O. Aboh

**Affiliations:** Amsterdam Center for Language and Communication (ACLC), University of Amsterdam, Amsterdam, Netherlands

**Keywords:** code-mixing, universal multilingualism, executive functions, hybrid grammars, recombination, syntax

## Abstract

In the literature, the term code-mixing/switching refers to instances of language mixing in which speakers/signers combine properties of two or more languages in their utterances. Such a linguistic behavior is typically discussed in the context of multilinguals, and experts commonly focus on the form of language mixing/switching and its cross-linguistic commonalities. Not much is known, however, about how the knowledge of code-mixing comes about. How come any speaker/signer having access to more than one externalization channel (spoken or signed) code-mixes spontaneously? Likewise, why do both neurotypical speakers/signers and certain neuro-atypical speakers/signers produce structurally similar mixing types? This paper offers some answers to these questions arguing that the cognitive process underlying code-mixing is a basic property of the human learning device: *recombination*, a fully automated cognitive process. Recombination is innate: it allows learners to select relevant linguistic features from heterogeneous inputs, and recombine them into new syntactic objects as part of their mental grammars whose extensions, arguably individual idiolects, represents what Aboh (2015b,a, 2019b) characterizes as hybrid grammars.

## Introduction

Over the past decades, there has been an increasing interest in multilingualism and its implications for the study of language, language use, and cognition. Nevertheless, it is not exaggerated to say that most formal approaches to language, language acquisition, and language change still regard multilingualism as exceptional, and thus rely on idealizations of monolingualism embedding a specific target uniform to every Speaker/Signer-learner (henceforth S-learner) of a community. Such frameworks are not conceived to model the linguistic knowledge of S-learners in highly multilingual communities, which nevertheless are very common. To wit, let us consider the background of this author, who grew up in a town in the South of Benin (West Africa) called *X*
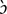
*gbónù* in Gungbe (Kwa), the native tongue of his father, *Àjàc$´\varepsilon$* in Yoruba (Benue-Congo)^1^, of which he has some basic knowledge, and Porto-Novo in French (Romance)^2^, which he speaks natively, albeit the *Béninois* variety. These three names are indicative of the major communities of speakers or languages in contact in this city of about 300,000 inhabitants. Though this author spent his adolescent and pre-adult years in *X
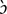
gbónù*, he was not born there but in Parakou, a city in the Northern part of the country. There, the major communities and languages in contact are Baatonu (Gur); Dendi (Songhai), and Waama (Gur). He does not speak any of these languages, though some of his siblings who were already attending school then do. At the age of six, his parents relocated in Agbomey, in the central part of the country. Here the major language is Fongbe (Kwa), which he speaks as L2. His family later relocated in *X
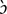
gbónù* when he was 11 years old. Finally, he has always been exposed to his mother’s native language Gengbe (Kwa), which he speaks only as heritage language. Then in secondary school, he learned English (age 12), and subsequently Spanish (age 16) which were obligatory in the curriculum and represented the so-called first and second “modern languages.” Working now in the Netherlands, he is exposed to Dutch of which he took lessons and has a passive knowledge.

This description shows that the linguistic knowledge of S-learners is in constant flux and so are the community networks generating the inputs S-learners are exposed to throughout life. This holds of speech communities in Benin, in Sub-Saharan Africa, and presumably in an ever-increasing number of urban zones in our globalizing world. Multilingualism is therefore becoming the standard, while pure monolingualism appears very exceptional.

Benin, the home country of this author, extends over 112,000 km^2^, with a population of about 10 million inhabitants who speak about sixty languages (excluding European languages). Given such a learning ecology, which we can take to be the norm for most S-learners in the world (and even more so in the Global South), several questions arise: Which language does such a multilingual S-learner speak natively? How can one define a native S-learner formally in such an ecology? How do the languages the S-learner knows interact during comprehension and production? What role do these languages play in subsequent learning experiences? What do the mental grammars of such an S-learner look like?

These questions have already been raised in the literature, but in a monolingual framework. An implicit traditional assumption is that linguistic theory describes the knowledge of an ideal S-learner who lives in “a completely homogeneous speech-community” and “knows her language perfectly” (cf. Chomsky, 1965, p. 5). Despite allowing a tremendous progress in formal linguistics and cognitive approaches to language, this methodology is not ecologically valid because it idealizes the exceptional case, rather than the default. The notion of a “perfect” S-learner becomes obsolete when one considers multilingual communities, population movements, and migration which all contribute to creating mosaic speaker/signer’s profiles.

In this paper, I argue for a different perspective: universal multilingualism, every S-learner is formally multilingual because s/he entertains several mental grammars ranging from registers, dialects of the same language, to typologically and genetically different languages. S-learners live in heterogeneous communities involving individuals with different experiences. It is therefore unlikely that S-learners harbor monolithic mental grammars that are opaque to each other. As has been shown by the rich literature on cross-linguistic influence, the languages of multilinguals affect each other, and a prevalent practice in multilingual communities is code-mixing: a behavior which does not match with the ideal of a “perfect” S-learner assumed in traditional approaches.

In Sub-Saharan Africa, for instance, most speakers alternate between different languages daily or mix different languages in their utterances. Sentence (1), constructed by this author, illustrates code-mixing. In the remaining of this article, I refer to this speaker, whose background is described in the preceding paragraphs, as “polyglot A.”

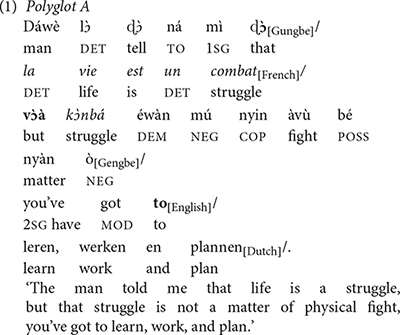

In this example, polyglot A combines pieces of structures from five different languages separated by the symbol “/.” These languages include Gungbe/French/Gengbe/English/Dutch in this order. Interestingly, the Gengbe stretch (i.e., the third sequence) contains a French loan word *k
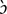
nbá* “combat.” Examples like these are characterized as code-mixing/switching in the literature and are typically assumed to require some cognitive capacity of the speaker. This polyglot, who is not an expert in all the five languages involved, could be thought of as being capable of deploying appropriate cognitive processes to select the switching point as well as the target grammatical categories in the relevant languages. Such a capacity, obviously, involves the ability to inhibit competing languages, for instance, the selection of French in the second segment rather than Dutch, or the selection of the Gengbe conjunction *v*
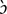
*à* rather than its competing equivalents in the other languages that this polyglot speaks (i.e., *but*_[English]_, *mais*_[French]_, *maar*_[Dutch]_, *àm*
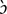
*n*_[Gungbe]_). Typically, polyglot A will use an utterance like (1) in a context in which the interlocutor also knows the five languages involved sufficiently to understand the utterance. Accordingly, polyglot A can also refrain from code-mixing (e.g., in a discourse context in which only one of the languages he speaks is allowed).

In contrast to (1) which might be thought of involving some “control” or language planning from its speaker, and could be representative of a neuro-typical cognitive phenotype, Fabbro (1999, p. 153–155) reports example (2) uttered by an aphasic polyglot, named E.G., a 55 years old and right-handed male. He spoke Slovene as mother tongue, Italian as L2, Friulian as L3, and English as L4. After a stroke, he exhibited Wernicke’s aphasia in all the languages he spoke, with “a severe mixing phenomenon in Italian, Friulian, and English” (Fabbro, 1999, p. 154). E.G. exhibited *pathological code-mixing*: In this example, E.G. combines English and Italian, regardless of the speech context. Aphasic patients showing pathological code-mixing cannot refrain from code-mixing. Throughout this paper, I present the data as reported in the sources.

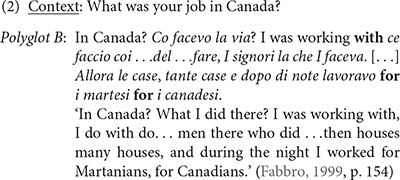

Despite the different conditions of their speakers, the examples in (1) and (2) show striking similarities with regard to their switching patterns: The switch occurs at clause boundaries, as indicated by the alternating languages in (1) or the sequence “In Canada? *Co facevo la via*?” in (2). In addition, the elements in boldface in these examples indicate that switching also occurs at the junction of grammatical elements such as coordinating and subordinating conjunctions as well as adpositions. It therefore seems that the cognitive process underlying the selection of the relevant categorial units of mixing is intact in both polyglots A and B. Observations like these, as well as comprehension data led Perecman (1989, p. 233) to conclude that “*the aphasic polyglot has an intact language system but imperfect control of the system* […] *the damaged brain processes language according to the same general principles as the non-damaged brain*” (see also Perecman, 1984 and references therein). One explanation suggested by Abutalebi et al. (2000, p. 54) is that what appear to be impaired in some aphasic patients showing pathological code-mixing are the inhibitory mechanisms responsible for deactivating lexical selection from the competing languages that the speaker has acquired.

If polyglots A and B only differ in their capacity to deploy the inhibitory mechanisms responsible for deactivating lexical selection, then the similar switch patterns in (1) and (2) which ultimately relate to structural properties call for a principled answer. What properties of the human language capacity explain these structural parallels? Why do speakers/signers (regardless of their cognitive phenotype) sometimes produce structurally similar mixing patterns even though they may be operating on typologically and genetically distinct languages (cf. 1–2)? What do such apparent structural commonalities tell us about the knowledge of code-mixing: the fact that any speaker/signer having access to more than one externalization channel code-mixes spontaneously, even if this linguistic behavior is not favored in her speech/signing community, and she has never been exposed to mixed inputs?

Current studies on code-mixing cannot answer these questions because they generally focus on the form of code-mixing, its social functions, and its cross-linguistic commonalities (e.g., Poplack, 1980, 2015; Myers-Scotton, 1998, Myers-Scotton, 2006; Muysken, 2000; MacSwan, 2005a, b; Kecskes and Albertazzi, 2007; Bulluck and Toribio, 2009). In addition, studies comparing properties of code-mixing between neuro-typical and neuro-atypical populations are sparse. Yet, answering these questions is essential to our understanding of the human language capacity, and how it is put to use in a multilingual context. Furthermore, understanding the fundamental similarities or differences between neuro-typical and neuro-atypical populations is important to establish which core aspect of the language capacity is resilient and presumably uniform to the species, and which aspect is less so.

In this paper, I take up this challenge and provide the first steps to answering these questions. I argue that the fact that the cognitive process underlying code-mixing in (1) is so entrenched in S-learners, appears to be very resilient, and prevails in absence of relevant language selection mechanisms (e.g., in some aphasic patients as indicated by example 2), suggests that it is a basic property of the human learning device: the language faculty. I show that this process, *recombination*, is present in all S-learners (monolinguals and bilinguals alike). During language acquisition, recombination allows S-learners to select relevant linguistic features from the heterogeneous inputs they are exposed to, and recombine them into pieces of mental grammars whose extensions represent individual idiolects, which Aboh (2015b) characterizes as hybrid grammars. In supposedly “monolingual” settings, hybrid outcomes of recombination are less noticeable because S-learners develop closely related variants. Yet, studies on the Flemish regiolect, the so-called *tussentaal* (De Caluwe, 2007; Ghyselen, 2016), as well as so-called ethnolects in various (urban) communities show that such mixes become apparent once the variants combined are more contrastive or involve typologically and genetically different languages (cf. the *International Journal of Bilingualism*, vol. 12, Issue 1–2, March 2008). I argue that recombination in traditionally assumed monoglots operates on closely related variants (e.g., registers or dialects of the same language), while, in polyglots, it involves more contrastive variants (i.e., typologically and/or genetically different languages). Based on these variants, S-learners develop an array of grammars that are combined during communication. This would mean that S-learners always operate in formally multilingual settings, that is, contexts in which different pieces of grammars (whether from dialects or registers of the same language or different languages) compete.

Section Universal Multilingualism and Code-Mixing discusses universal structural properties of code-mixing across neural-typical and neuro-atypical speakers/signers. These examples indicate that code-mixing emerges spontaneously as a result of *recombination*, an innate capacity.

Building on this, section Recombination: An Innate Capacity discusses the role of executive functions in language processing/production, and proposes a view of the Human Language Capacity in which recombination is fully automated, while selection of vocabulary items for spell-out purposes is mediated through executive functions. This would mean that surface manifestations commonly referred to as code-mixing, code-switching, code-blending, etc. only become apparent when some (aspects) of the competing languages of the polyglot are not inhibited so that several lexica are used to spell out a unique structure^3^.

Section Implications for the Study of Language further discusses the consequences of this framework for the study of language, including the common notion of “grammaticality judgment” which is redefined accordingly. The last section concludes the paper.

At this point, a disclaimer is in order. This paper is programmatic in nature: it raises fundamental questions about how to characterize the human mind through the lenses of universal multilingualism, the consequences that this view has for a linguistic theory based on linguistic hybridism, and how the assumptions made here relate to different subfields of linguistics. Accordingly, the discussion leaves out some technical syntactic details which I postpone for future work.

## Universal Multilingualism and Code-Mixing

This paper discusses aspects of code-mixing, also referred to in the literature as intra-sentential code-mixing. Adapting Muysken’s (2000, p. 1) definition, I use the term code-mixing to mean *all cases in which aspects of lexical items and*/*or grammatical items of different languages/varieties are combined into a single linguistic expression*^4^. In terms of this definition, and as already mentioned in section Introduction, examples (1) and (2) indicate that the neuro-typical and the neuro-atypical speakers behave similarly: their utterances involve comparable switching points:

(i) + ⁣/⁣− Finite complementizers (including prepositions),
(ii) Clause boundaries,
(iii) Prepositions (introducing adjuncts or new arguments).

Recall, however, from the introduction that polyglot A presumably falls within the spectrum of neurotypicality, and as such can control for the languages used in code-mixing or refrain from code-mixing in appropriate context. Polyglot B, on the other hand, exhibits *pathological code-mixing*: a condition in which some patients’ utterances involve “frequent and uncontrolled switching to another language” (Fabbro, 1999, p. 142). As the data from E.G. – the speaker of example (2) – show, such patients cannot inhibit the competing languages they speak.

The literature on aphasic polyglots showing pathological code-mixing includes very many reports indicating that such patients produce mixing patterns that fall well within the general typology of code-mixing (cf. Perecman, 1984, 1989). Following Muysken’s (2000) typology, example (2) instantiates *alternation* between Italian and English. Example (3) reported in Chengappa et al. (2004, p. 71) instantiates *insertion*: a lexical element or a constituent from one language is integrated in another language. All participants in Chengappa et al. (2004) study suffered from Broca’s aphasia in both Malayalam and English. Example (3a) represents a Malayalam context, while (3b) illustrates an English context.

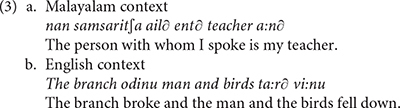

This study further reports that some patients can engage in what Muysken (1981b) defined as *relexifiation*: a cognitive process by which speakers spell out the grammar of one language drawing on lexical items from a different language (cf. Mous, 2003). Such examples are given in (4), which the authors argue are built on the Malayalam equivalents suggested below each sentence.

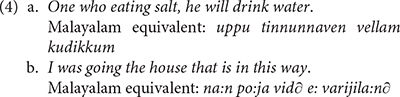

The examples in (5) show that the patients in this study also produced word-internal mixing, as clearly indicated by the Malayalam affixes combined with the English words.

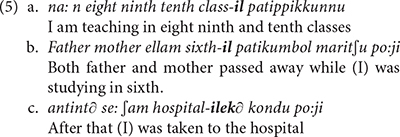

Such word internal mixing has already been reported in the literature, and appears to be very systematic with regard to the selection of affixes and roots combined. The process does not seem random: the affixes generally match the category of the root they attach to, nominal affixes attach to nominal roots, verbal affixes combine with verbal roots, etc. For instance, E. G., who uttered example (2) discussed above, also produced words in which an Italian affix −*a* was attached to an English noun root, as illustrated by *carra* in (6a) (cf. Fabbro, 1999, p. 155). Examples (6b) and (6c), also discussed by Fabbro (1999, p. 155, 156), involve productions of German-English bilinguals which instantiate combination of a verbal affix from one language with a verbal root from another language. Finally, example (6d) taken from Perecman (1984, p. 51) is produced by a patient, named H.B., who was asked to interpret the phrase *a swelled head*^5^. While the translation he produced did not match the English equivalent (e.g., *head* is translated as *haus* “house”) his Germanicized English root *∫vεldεs* is combined with a Germanic past participle affix *ge*− to form ***g***ε-*∫*νε*ldεs*.

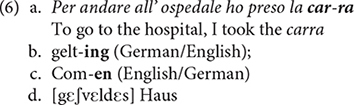

The examples presented here have all in common that they represent morpho-syntactically well-formed outputs of the types commonly observed in situations of language contact or “language creation” within neurotypical populations in which word categories of one language are combined with relevant grammatical elements of another language, as instances of what Aboh (2015b) refers to as hybrid constructs (see also Mufwene, 2001, 2008; Aboh and DeGraff, 2014, 2016, and references therein). Indeed, the examples of word-level mixing presented here do not represent “illicit” syntactic units involving, for instance, a combination of a nominal plural affix with a future auxiliary, or a gender morpheme with an aspect marker, etc. The patients who produced the forms discussed here show uncontrolled language mixing, and one could think that this would also affect lexical selection across their languages, such that any grammatical element or morpheme in one language can randomly combine with any other element in another language. This is, however, not the case in the data discussed here. These speakers produce perfectly “licit” syntactic objects which, in favorable circumstances, can be conventionalized into a community language. It therefore seems that the cognitive process responsible for the selection of relevant grammatical categories is intact in these patients.

Indeed, nothing distinguishes formally between the examples in (3)–(6) and classical cases of code-mixing in neurotypical populations discussed in the literature (cf. Muysken, 2000 and references therein)^6^. This is clear from the following Media Lengua example discussed in Muysken (1981b).

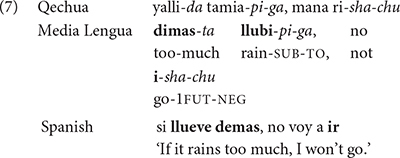

In this example, the content lexical elements in boldface are taken from Spanish, while the grammatical items in italic are selected from Qechua. Similar examples involving various languages abound in the literature, and nothing distinguishes them formally from those in (6), though the latter are produced by neuro-atypical speakers. We can therefore conclude from these facts that the cognitive capacity underlying code-mixing is sensitive to categorial distinction involving grammatical features, and appears to generate well-formed syntactic objects only. Accordingly, I assume this extremely resilient cognitive capacity to be innate and therefore uniform for all S-learners.

This assumption is further supported by bimodals, that is, individuals who acquire a spoken and signed language natively (e.g., hearing children of deaf adults, cf. Bishop and Hicks, 2005, 2008, and references therein). Data from these bimodals represent strong evidence that code-mixing emerges spontaneously since the inputs these S-learners are exposed to are unimodal (either spoken or signed). The example in (8), reported in Donati and Branchini (2013) and Branchini and Donati (2016), illustrates bimodal code-mixing in Italian and Italian Sign Language (LIS). In this example, the first line represents Italian, while the third line includes constituents in LIS. The second line shows the gloss and the columns group together constituents which are spoken and signed simultaneously.

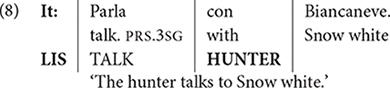

Similar examples are reported in İşsever et al. (2018) for Turkish and Turkish Sign Language, and Emmorey et al. (2005) for English and American Sign Language. These facts show that while code-mixing is sensitive to structural properties, its spellout need not be sequential, as one could believe looking at spoken languages only (cf. Donati and Branchini, 2013).

Like with polyglots in spoken languages, bimodals too can exhibit pathological code-mixing between the two modalities. Fabbro (1999, p. 152) reports the case of a patient who prior to insult could accompany his signs with individual words in other languages he had learned (e.g., Czech). After insult, however, this patient lost this capacity and produced combinations of signs and unrelated spoken words.

### Interim Conclusion

We can conclude from this discussion that the cognitive process underlying code-mixing is extremely resilient, and appears present in all humans who possess language. The discussion further shows that code-mixing is rule-governed regardless the “neuro-phenotype” of the S-learner (cf. Perecman, 1984, 1989). This would suggest that even though the capacity to code-mix is present in all S-learners, mono-lingual/modal and multi-lingual/modal alike, it may go unnoticed when speakers/signers operate on closely related vocabulary items.

These conclusions raise two puzzles:

(1) Speakers/signers code-mix spontaneously: Where does this capacity come from?
(2) Limits of syntactic computation: Structural similarities between mixed outputs of neuro-typical and neuro-atypical speakers/signers strongly suggest that the human mind does not produce formally illicit (or ungrammatical) outputs, that is, structures outside the range of Universal Grammar. If so, how can we further understand the notion of “ungrammaticality”?

According to Chomsky (1995, p. 14), the human language capacity can be conceived of as a continuum involving an initial state, which is innate and “uniform for the species” and a final state that results from the experience of the S-learner. Universal Grammar (UG) represents the theory of the initial state. In terms of Chomsky (2005, p. 4):

UG must provide, first, a structured inventory of possible lexical items that are related to or perhaps identical with the concepts that are the elements of the “cognoscitive powers,” sometimes now regarded as a “language of thought” […]; and second, means to construct from these lexical items the infinite variety of internal structures that enter into thought, interpretation, planning, and other human mental acts, and that are sometimes put to use in action.

In the context of this definition, the data of code-mixing from neuro-typical and neuro-atypical speaker’s profiles discussed in this paper suggest that the notion of *ungrammaticality* formally involves two aspects: one that relates to non-converging outputs (i.e., illicit outputs filtered out by UG) due to constraints on the computational system, and one that relates to conventionalized forms in the lexicon (i.e., illicit outputs within a speech community). The latter relates to acceptability judgments offered by S-learners of a specific variety.

## Recombination: an Innate Capacity

In addressing the facts presented in section Universal Multilingualism and Code-Mixing and the puzzles they raise, I make three working hypotheses, which I now discuss in turn:

*Working hypothesis 1*: The cognitive process underlying code-mixing is what drives acquisition. This hypothesis is based on the fact discussed in section Universal Multilingualism and Code-Mixing that S-learners demonstrate the capacity to code-mix spontaneously, even if they live in a community in which such linguistic behavior is not favored, and could not be said to be part of their learning experience (e.g., bimodals). Code-mixing therefore appears to be contingent upon acquisition of language. The discussion has also shown that not only is the capacity of code-mixing present in all S-learners regardless of their cognitive phenotype, but the outputs within and across populations share striking structural similarities. Put together, these facts indicate that S-learners have an instinct for code-mixing: an innate capacity. S-learners are born endowed with the capacity to code-mix.

*Working hypothesis 2*: Vocabulary selection (as understood in formal syntax) is mediated through the executive functions. In accounting for pathological code-mixing, Abutalebi et al. (2000, p. 54) conclude that this condition is not due to language processing or code-mixing *per se* but to a dysfunction in the executive function system, “*the control mechanism subserving lexical selection across languages*” (see also Perecman, 1984; Fabbro, 1999; Paradis, 2004; Abutalebi and Green, 2008; Green and Abutalebi, 2008). This conclusion is compatible with the view developed here that the capacity of code-mixing must be dissociated from executive functions responsible for lexical selection. *Executive functions* is a cover term for various cognitive processes involving attention control, behavioral inhibition and working memory, all necessary for the deliberate control of goal orientated actions (cf. Gooch et al., 2016). Several studies report an interaction between executive functions and vocabulary learning, and hence language acquisition (e.g., Kalia et al., 2017).

*Working hypothesis 3*: If code-mixing is innate and drives acquisition but is subject to the executive functions for vocabulary insertion, then the cognitive process which produces code-mixing, that is, recombination, must precede vocabulary selection. Executive functions are necessary for the selection/learning of a specific lexicon or vocabulary, but they must be deployed after syntactic computation.

**FIGURE 1:**
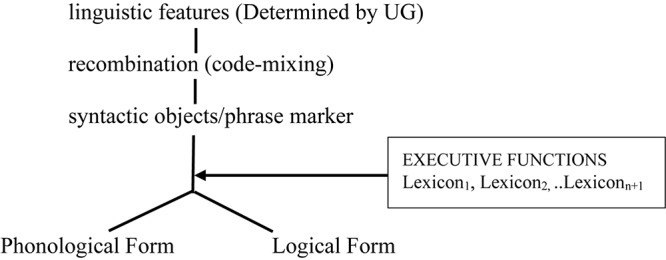
A tentative model of grammar.

Together, these three working hypotheses lead to the tentative model of grammar, based on the generative traditional “Y-model,” as represented in Figure 1.

This model is compatible with the view that some surface phenomena (e.g., affix reordering) are post-syntactic (as commonly assumed in Distributed Morphology, cf. Halle and Marantz, 1993, 1994, and much subsequent work). Under this view, such surface phenomena happen when executive functions are deployed, that is, after the phrase marker has been built. This view seems supported by instances of pathological code-mixing at the phonological level (e.g., the pronunciation of a vocabulary of one language with the intonation of another), reported in Perecman (1984, 1989)^7^. Though the issue is not uncontroversial (see for example, Tsiplakou et al., 2016; Leivada et al., 2017; Alexiadou and Lohndal, 2018), MacSwan (2005a, p. 5) claims that code-mixing at the phonological level is ruled out under his *PF Disjunction Theorem* (see also MacSwan, 2005b for discussion). Space limitation prevents me from exposing his arguments here, but the relevant point is that the tentative model in Figure 1 is compatible with the observations in the literature: the apparent absence of code-mixing at PF in neuro-typical populations, but not in neuro-atypical ones. In this model, executive functions intervene after recombination, but before PF, hence there may be no code-mixing once lexical items are selected with their related PF-ordered rules. MacSwan’s (2005a, b) PF filter bans word-internal mixing which does occur, as already discussed in the literature, and as the data surveyed here (e.g., examples 3, 6) further attest to. In an approach to mixing based on Distributed Morphology, Alexiadou and Lohndal (2018) argue that bilinguals have access to a default mechanism that allows the integration of roots from one language to the morphology of another (e.g., German roots to Greek morphology)^8^. According to these authors, “the bilingual speaker in view of the fact that she has more [vocabulary items] at her disposal will pick an overt realization, if a default such realization is available. The default realization is the one that is compatible with the largest number of roots, i.e., the roots of both languages.” (p. 11). They further conclude: “if speakers can pick among different types of n/v to combine with roots, they pick those that will fit the general phonology/properties of the phase head. Put differently, the phonology within a phase head needs to be uniform.” (p. 12). Rather than a general ban on word-level mixing, this phase-level PF-filter offers a more parsimonious analysis of word-internal mixing and appears compatible with the framework developed here based on recombination and hybrid grammars.

The discussion in this section also indicates that recombination (the capacity to combine morphemes into larger well-formed lexical items) remains intact even when the executive functions are obliterated. Accordingly, this resilient cognitive capacity which allows S-learners to select relevant linguistic features from the inputs and recombine them in new linguistic objects can be assumed to be innate, and forms part of the human “instinct for language^9^.”

In this approach, recombination is an innate, fully automated, cognitive capacity. It is an instance of general MERGE (as defined in Chomsky, 1995) applied to linguistic features relating to different components of the grammar (phonology, morphology, syntax, semantics). I have already mentioned that both monoglots and polyglots exhibit recombination, but differ with regard to the variants that the process operates on (Aboh, 2015b). Recombination in the “monoglot’s mind” is restricted to closely related variants (i.e., of the same language or dialect), while recombination in the “polyglot’s mind” may operate on distant variants (i.e., from different typological and/or genetic languages). That recombination is contingent on acquisition is also supported by the fact that the process generates licit syntactic objects even in the absence of a “coherent” lexicon. Recall, for instance, the examples of word-level mixing shown in (6), and repeated in (9) for convenience^10^.

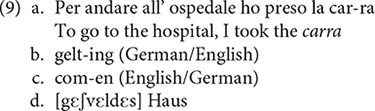

Though much study is needed to fully understand the interaction between recombination, the derivation of clause structure, and the interfaces with PF and LF, the model proposed here suggests that the language faculty is a much more dynamic and flexible system than commonly assumed. This view is in line with recent advances in neurosciences indicating that language processing relates to a diffused combinatory network in the brain (e.g., Vigliocco, 2000; Kaan and Swaab, 2002; Friederici, 2011).

While I hope to return to these questions in future collaborative work, an urgent question now arises: How does a model advocating universal multilingualism relate to acquisition by monoglots (or acquisition *tout court*)? Answering this question has some implications for the study of language, language acquisition and change, which I will elaborate on further in the following sections.

## Implications for the Study of Language

The discussion in previous sections shows that speech communities are heterogeneous in terms of their linguistic practices (i.e., not all members of a community develop exactly the same competence in all registers/dialects/languages in the community). In this regard, the remarkable versatility of S-learners in language use suggests that Roeper’s (1999) notion of formal bilingualism, should be understood as *formal multilingualism*, the null hypothesis in any learning setting.

### Variation Within and Across Individuals

Every S-learner is exposed to a range of variants, that are arguably expressions of different language types (or different grammars). This appears obvious in a context like Benin, as depicted in the introduction, but it can also be shown for speech communities which are not typically assumed to be multilingual. It is, for instance, common practice to focus on Standard French in studying acquisition of French. Yet, a probe into individuals shows that while speakers may all converge in producing the following three grammatical options to express direct yes-no questions, these constructions do not have the same distribution nor do they have the same status (cf. Arrighi, 2007; Dagnac, 2013).

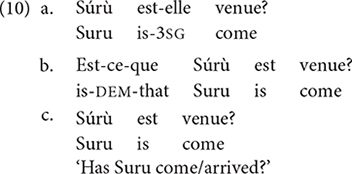

Though these three strategies have the same meaning “has Suru come/arrived?”, each has a very different status: (10a) appears in texts mostly and represents a high register. (10c) is the most common strategy typically used in spoken French because it makes use of intonation only, and (10b) has an intermediary status appearing both in formal and informal contexts. Yet, these three strategies which are typically analyzed in terms of register, relate to yes-no question formation strategies found in typologically different languages. (10a) can be said to be typical of Romance, with clitic inversion. (10b), analyzed as involving a question marker *est-ce-que*, is comparable to languages involving a sentence-initial question particle added to a simple declarative clause. (10c), with final rising contour added to a declarative clause, is comparable to languages in which a sentence-final question particle (sometimes a tone, such as in Gungbe) is added to a simple declarative clause (see Dryer’s, 2013, description in WALS for some typological distribution). These three strategies relate to three pieces of grammars found cross-linguistically. Accordingly, contemporary French speakers internalize three typologically different pieces of grammars in their expressions of yes-no questions.

The traditional generative approach to such systematic variation, would be to assume that these three separate registers are somehow part of a holistic mental grammar (in which competing variants are sometimes filtered out, see Roeper, 1999, for a critique). Yet, the impressive range of variation within and across individuals, the magnitude of the variants an individual can harbor as well as the flexibility with which S-learners adapt to, and adopt new variants used by their interlocutors suggest that such a view cannot be correct. If it were, there would be much less variation within and across S-learners and languages than there actually are. The dynamicity of human linguistic capacity suggests otherwise, and so do sociolinguistic studies, since Labov’s seminal work in the 60s, which show how systematic S-learners are in combining and using variants they are exposed to, while creating new ones (see for instance, Doğruöz and Backus, 2009, Backus, 2010; Ghyselen, 2016; Ghyselen and De Vogelaer, 2018, for some recent references). Likewise, work on diachronic changes (e.g., Kroch, 1989, 2001, Lightfoot, 2006, and much related work), indicates that S-learners may entertain different competing grammars, even in the same language.

While I maintain that linguistic features compete in the mind of the S-learner (cf. Aboh, 2009), I further propose that what S-learners internalize is a rainbow of pieces of grammars (such as in 10) that are put together in communication. The central point here is that learning feeds on heterogeneous inputs that are in a state of flux, and the outputs of recombination are hybrid mental grammars (Aboh, 2015b, 2019a). The proposed view in terms of recombination as a fully automated cognitive process, independent of lexical insertion, leads me to conclude that acquisition of a language (i.e., a conventionalized system used in a speech community) boils down to deploying the executive functions to map the outputs of recombination on specific lexica.

**FIGURE 2:**
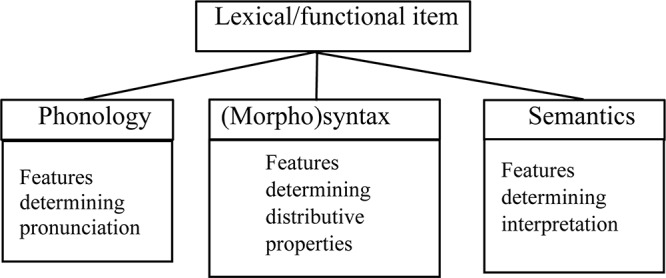
Lexical/functional item.

In this regard, I (Aboh, 2015b, 2017, 2019a) explain the role of recombination of linguistic features in the emergence of bundles of features that are mapped onto specific lexical items, in contact situations that led to the emergence of creole languages^11^. The demonstration is based on the Minimalist assumption that a lexical item embeds three components minimally: phonology, morphosyntax, and semantics (cf. Figure 2).

**FIGURE 3:**
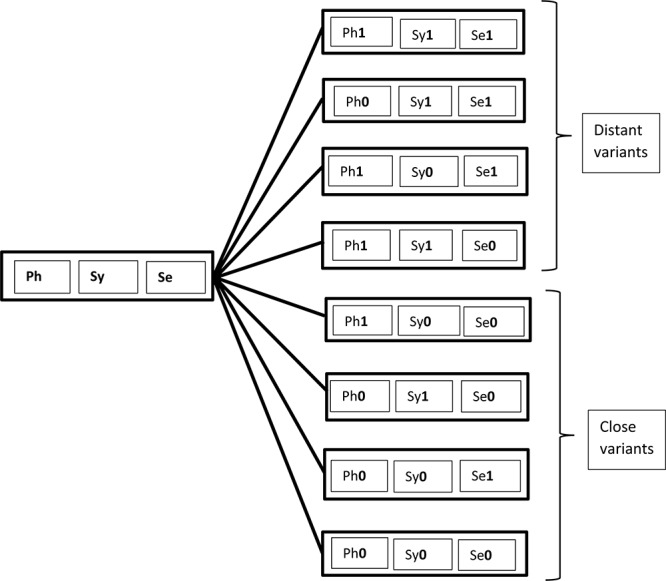
Possible combinations in learning a lexical item.

I argue in these studies that features pertaining to these three components can be recombined individually during acquisition, based on the learner’s hypotheses over the inputs she is exposed to. Recombination is responsible for variation within and across individuals because S-learners develop different versions of the bundles of features mapped onto specific lexical items (cf. Alexiadou and Lohndal, 2018). In principle, every acquired lexical item relates to seven potential competing variants. Consider Figure 3 (taken from Aboh, 2017, 2019a,b) in which the digit 0 represents the target language, while 1 represents a point of change.

This figure shows potential variation in the inputs due to S-learner’s approximations. Aside perfect replication (i.e., the box with three digits “0” at the bottom), which no one, or maybe only a few experts achieve, learning may generate seven other competing variants. The figure also indicates that variants created by S-learners approximate the target to various degrees. A variant which exhibits semantic change only (e.g., the second box from the bottom) is closer to the target than one that involves a phonological, a morphosyntactic, and a semantic change (e.g., the upmost box). Though these variants arguably form a continuum, they can be described in terms of two classes. The first class, referred to as “close variants” in Figure 3, involves variants which have modification in one component only, and are arguably close enough to the target to go unnoticed within the community or to be tolerated as possible variants. For example, a lexical item characterized as (Ph1, Sy0, Se0) can be labeled as a “different accent” by speakers of a community (e.g., speakers from Newcastle are generally considered native speakers of English even though they do not sound like speakers from London, and *vice versa*).

The second class includes outputs that I refer to as “distant variants.” Similarly to instances of recombination observed in pathological code-mixing (cf. 9), these variants are licit grammatical options. However, they may be farfetched from speech conventions in a community, and speakers/signers may consider them to be marginal, degraded, or even unacceptable. Nothing, however, prevents such variants from competing with “close variants” and spreading within a community if circumstances permit. This is how we can account for the variation between Standard American English adverb *rather* as in (11a) versus instances of verbal *rather* (11b-c), which Wood (2013) shows is part of the grammar of some speakers of colloquial American English.

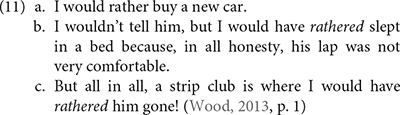

The same could be said of modal combinations, which are generally assumed to be ungrammatical in Standard American English, but which have been shown since the 60s to occur in many varieties of Southern American English, as illustrated by (13) taken from Mishoe and Montgomery (1994, p. 9–10)^12^.

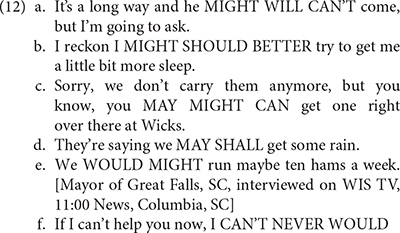

How such variants arise in the mind of S-learners (partly based on the inputs they are exposed to) can be illustrated by the following examples from my Béninois French. In this variety, it is perfectly acceptable to utter the sentence in (13) which includes three instances of the verb *manger* “eat” and whose approximative English translation is given below.

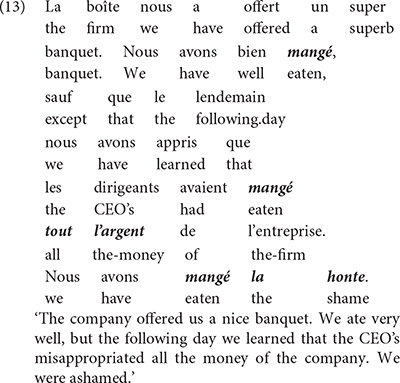

The first instance of *mangé* obeys the morphosyntax of this verb in standard French, in which it can also be used intransitively as *eat* in English. The second instance, however, appears a bit distant from the French standard usage. In this case, the verb *manger* is combined with *tout l’argent* “all the money” to mean misappropriate all the money. At this point, one could imagine that this construction is a mere metaphorical use of the verb *manger*, comparable to French idiomatic expressions such as *manger la consigne* (lit. eat the recommendations; “ignore/forget the recommendations”). First, it is important to realize that such French idiomatic expressions are not in the inputs of most Béninois speakers (I had to look this one up in a dictionary, and everyone I asked around me in Benin did not know this expression, and could not even guess its meaning). Second, Béninois French allows a third instance, *mangé la honte* (lit. eat the shame), to mean to be ashamed. Other similar constructions in Béninois French involve *manger la vie* (lit. eat the life) to mean enjoy life. Therefore, the usage of *manger* with non-consumable abstract object DPs to form expressions with unpredictable meanings appears much more productive in Béninois French, than it seems in Standard French. Aboh (2009, 2015b, 2017, 2019a) show that such expressions derive from a combination of Standard French with properties of Inherent Complement Verbs (ICV) found in Gbe and many Kwa languages (cf. Essegbey, 1999; Aboh, 2015a). ICVs are verbs which in their citation form require an accompanying object in the form of an NP. The translations of *manger*, *spend* or *be ashamed* in Gungbe involve a verb of this class in which the verbal element *ù* combines with an NP, as indicated in (14).

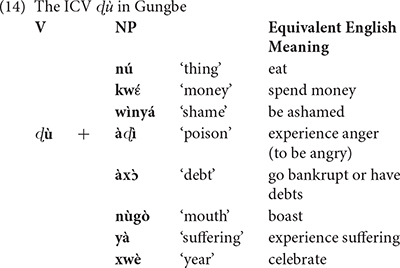

The variety of meanings in (14) indicates that the verbal element **ù** does not seem to have a clear meaning on its own, since it must combine with various NPs to form different meanings, hence *ù* + *nú* “thing” translates as “eat,” **ù** + *kw*\´textepsilon “money” translates as *spend* and **ù** + *wìnyá* “shame” translates as “be ashamed,” etc. It is these meanings that are incorporated in French *manger* in example (14). The lexical item *manger* in the idiolect of this speaker can then be described as in (15).

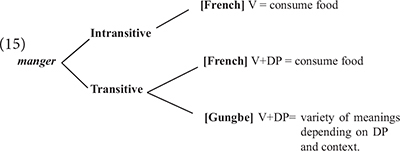

Aboh (2015a) argues that ICVs involve a functional verb which first merges in *v* unlike lexical verbs which merge in V. Comparing *ù nú* in Gungbe to *manger* in French, we reach the contrast in (16a) for Gungbe versus (16b) for transitive *manger* in French.

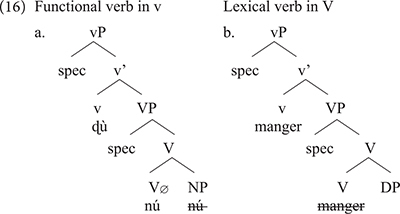

The two languages differ in a number of respects: *manger* in Standard French being a lexical verb, it merges in V where it selects a relevant DP. This is different from Gungbe in which the lexical verb is null but has categorial requirement on its bare NP-complement, here *nú* which further incorporates in V. The lexical verb raises to *v* in French, but this movement is impossible in Gungbe in which *v* contains the functional verb *ù*, which introduces the external argument. Based on examples such as *ù* + *nú* “V + thing” in which the complement NP *nú* is a dummy element, Aboh (2015a) concludes that such a functional verb only encodes that the external argument has some experience/relation with the set referred to by the complement bare NP. The nature of this experience/relation is inferred from the context. This is why the meaning of the verbs in (14) is not compositional and cannot be entirely predicted based on the NP complement. I refer the interested reader to Aboh (2015a) and references therein for further discussion on ICVs. What matters, however, for the present discussion is that the usage of *manger* in the expressions *manger tout l’argent* and *manger la honte* in example (13) results from the integration of structure (16a) into French. These expressions involve a functional usage of the verb *manger* which first merges in *v*, while V has no phonological content. Note, however, that the combination of Gbe and French yields a new empty V that selects for a full DP, hence the occurrence of the quantifier *tout*, and the definite determiners *le*/*la* in these examples (17).

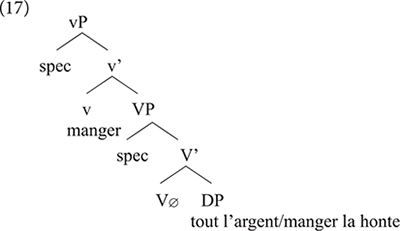

Recombining properties of *manger* in French to those of ICVs in Gbe, and therefore coining a functional verb *manger* in Béninois French, leads to a new structure not found in the two languages. This, in turn, is a point of change between Standard French and Béninois French. In the new structure V_∅_ has no phonological content, and it is not spelled out because its complement, a DP or QP blocks N-to-V incorporation, unlike bare NPs in Gbe (or Kwa in general). Structure (17) can therefore generate *manger tout l’argent* or *manger la honte*; but not ^∗^*manger argent* or ^∗^*manger honte* which would be perfect replicas of Gungbe as in (16a).

*Manger* in French arguably spells out two nodes within the vP: v-V. However, in the Béninois French usage of *manger* in a way similar to ICVs, this lexical item only spells out v, leaving V unpronounced. This suggests the description below.

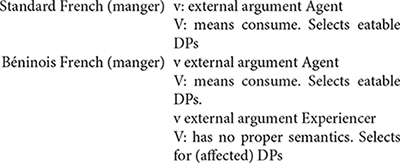

### Recombination, Grammaticality Judgment, and Limits on Variation

The discussion in previous sections shows that recombination accounts for S-learners’ variation as resulting from the acquisition of the lexicon, and sheds new light on the notion of acceptability judgment as a formal notion central to the inquiry of S-learners’ competence (Chomsky, 1965). Figure 3 indicates that the common notion of “grammaticality” involves two aspects. One, understood as acceptability, relates to the lexicon and can be defined as the conventions allowed within a speech community. For instance, there is no computational or UG principle that bans verbal *rather* (11) in American English or functional verb *manger* in French (13). Though excluded from the pool of “close variants” in Standard American English and Standard French, respectively, these “distant variants” (cf. Figure 3) nevertheless represent well-formed linguistic objects involving specific bundles of features. Studies investigating this type of acceptability are only informative to the extent that they expose the conventions at work within a particular speech community. Consequently, so-called grammaticality judgment tasks that tap into S-learners’ knowledge of such conventions do not directly inform us on the constraints on the computational system which may translate into constraints on linguistic variation. By tracking such conventions within a community, we actually gather knowledge on E-language, and may not immediately deduce any broad generalization about the human language capacity unless we take a broader comparative typological perspective that can help identify gaps, which in turn inform us on possible constraints on the language faculty.

The present discussion on recombination may give some readers the feeling that any combination is possible, yet this cannot be true, as clearly indicated by the relatively small number of structural types discussed in typological books. This makes sense if variation of the structural type is constrained by properties of the computational system. This brings us to the second side of grammaticality: which relates directly to the limits of the computational system. Violations on principles of the computation (e.g., Minimality effect, feature mismatch) hold universally, but they are extremely difficult to investigate (as any fieldwork linguist would recognize). For instance, we saw in previous sections that aphasic patients showing pathological code-mixing produce patterns which, even though unacceptable from the point of view of a specific lexicon, are well formed syntactic objects sometimes conventionalized in so-called new languages (e.g., mixed languages). Accordingly, constraints on the computational system can only be studied experimentally or through introspection, rather than based on naturalistic data or corpora. If we consider the role of Minimality in recombination, for instance, I’m not aware of any instance of non-local recombination^13^ in neuro-typical populations engaged in creative language use in which an affix may be recombined across an intervening affix (e.g., recombination of an affix and a root across another blocking affix, as in greed-ness-y for greed-i-ness). I’m also not aware of speech errors involving such Mimimality violations (cf. Pfau, 2009). It therefore seems that Minimality violating (or formally “ungrammatical”) recombination is generally absent in spontaneous productions of neuro-typical populations. Minimality, therefore, is a strict condition on the computational system. The interaction between grammaticality constraints on specific lexica and formal constraints on the computational system yields the range of variation observed cross-linguistically, as well as the strong commonalities that human languages display. These two levels of grammaticality are not always systematically distinguished in the literature, sometimes leading to confusions or misunderstandings as to the relevance of naturalistic data versus controlled experimental data. In this paper, grammaticality over the lexicon informs us on the contours of patterns on the population level and how that relates to some conventionalized forms in a specific lexicon. Grammaticality over recombination informs us on limits of the computational system itself, that is, what is humanly possible, and arguably learnable.

### Constraints on Variation

In this regard, Aboh (2015b, 2019a) reports a fact discussed in the literature since the early 80s by typologists as well as creolists (e.g., Bickerton, 1981, 1988; Muysken, 1981a; Foley and Van Valin, 1984; Baker, 1985; Bybee, 1985; Hengeveld, 1989) and further formalized recently by Cinque (1999) within the cartographic framework: All human languages described to date display a fixed ordering of Tense, Mood, and Aspect (TMA) expressions as schematized in (18a). In this schematic sequencing, each label stands for a more articulate domain involving distinct tense, mood, or aspect expressions. An illustrative Gungbe sentence is given in (18b), whose sequencing is described in (18c) (cf. Aboh, 2004).

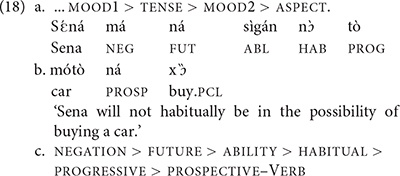

Cross-linguistic studies have shown that this sequencing or some variant thereof spontaneously emerges in new languages (e.g., creoles). Likewise, even though such TMA expressions can precede or follow the lexical verb and may display different derived orders, the scope hierarchy in (18a) is always maintained such that no example of random combinations or reordering (e.g., aspect markers being further away from the verb than epistemic modals) has been described for any human language (cf. Baker, 1985; Hengeveld, 1989, 2006; Cinque, 1999; Ramchand and Svenonious, 2014). This is so, even though languages may show extreme morphological variations as to how to express each label, some using affixes, other resorting to free morphemes, while others even use tones. Given our previous observations about recombination and limits on computation, it seems reasonable to assume that the absence of cross-linguistic structural variation within the so-called INFL domain is due to Minimality. TMA elements can only be recombined locally, that is, only adjacent heads can recombine and recombination cannot operate across an intervening head (i.e., a TMA). Accordingly, an aspect head (e.g., expressing progressive in 18b) cannot be merged directly to the future head, across the habitual head and the ability head. This is so, even though progressive tends to be used to encode future time reference in many languages, as in *I’m gonna leave* in English. Note that, in this example, however, recombination happens between *going* and *to*, which are arguably adjacent in the derivation. Minimality therefore severely constrains structural reordering patterns within the TMA, hence the astonishing uniformity observed cross-linguistically. The current discussion indicates that human languages are structurally alike with regard to their TMA domain. The question now arises whether this uniformity applies to other structural domains as well, or whether there are loci of structural variation which may be the core of typological variation.

Not much is known about this question as there is not yet a typology of the points of structural variation within human languages. In this regard, Aboh (2019a) reports that the cross-linguistic stability observed within the TMA domain, does not seem to immediately carry over to the CP domain, which Rizzi (1997) analyses as involving the schema in (19). Force encodes clause-typing, Inter expresses interrogative features, Top hosts topic elements, Foc licenses focus phrases, and Fin realizes finiteness properties of the embedded TP.

(19) ForceP…InterP…TopP^∗^…FocP…TopP^∗^…FinP… TP…VP.

Much work is still necessary before we have a better insight into cross-linguistic variations within the CP-domain. Yet, a cursory look at the existing literature on Information Structure (IS), and its related word order patterns, largely determined by the CP-domain, indicates that IS is the source of sharp cross-linguistic structural variations. Starting with commonly studied languages, a naïve look at Slavic versus Germanic and Romance, shows that while the former can be said to exhibit morphologically rich agreement patterns, these does not make them particularly striking compared to the latter. Instead, what makes Slavic languages stand out typologically is the intricate relation they display between IS and Syntax, which led experts to label them as discourse-configurational languages (e.g., É-Kiss, 1995 and references therein). Germanic languages, however, are well-known for exhibiting V2 phenomena, virtually absent in Romance, for instance. With regard to Niger-Congo, many studies reveal that most languages of this family exhibit a rich set of discourse markers that realize the clausal left periphery and mark discourse-related constituents such as topic and focus. Such left peripheral markers are not typical of Slavic, Germanic, and Romance which rely more on word order and intonation for IS purposes. Indeed, discourse markers in Niger-Congo typically trigger displacement operations which sometimes result in a whole sentence being pied-piped to some left peripheral position (cf. Nkemji, 1995; Aboh, 2010, 2016). Note, however, that heavy pied-piping for the purpose of discourse is not pervasive in Slavic, Romance or Germanic languages.

There is also significant variation within language families. For, instance, while some Romance languages (e.g., Italian) allow recursive topic phrases to precede and follow a unique focus projection (Rizzi, 1997), others (e.g., French) exclude such structures. Within Niger-Congo, some languages display *ex situ* wh-movement only (e.g., Gbe), others involve both *ex situ* and *in situ* strategies (e.g., Gur, Bantu). Finally, some Germanic languages display superficial V3 patterns, while others exclude such sequences. Discussions on IS and its relation to the clausal left periphery therefore suggest that languages tend to vary more structurally within this domain.

Accordingly, the structural rigidity that prevails within the TMA domain does not seem to hold when it comes to the clausal left periphery: the CP-domain. Under the reasonable hypothesis that the licensing of discourse markers, V2, and wh-phrases are all properties of specific heads within the CP-domain, it appears that the range of structural variation within this domain is more pronounced than originally assumed. Finally, there is a wealth of literature on language acquisition within different S-learner profiles (e.g., L1A, L2A, Heritage learners, learners with Developmental Language Disorder) showing that while (advanced) S-learners may be target-like with regard to properties of the TMA domain, they may experience more difficulties with IS-related constructions (Haznedar and Schwartz, 1997; Lardiere, 2000; Prévost and White, 2000; Goad and White, 2004; Sorace, 2005; Tsimpli and Sorace, 2006; Sorace and Serratrice, 2009; Polinsky, 2018). Accordingly, there appears to be a fundamental asymmetry between the CP-domain and the TMA domain.

Translating this asymmetry in phase theory (Chomsky, 2000, 2001), let us assume the label L to be a shorthand label for all left peripheral phases, including the clause and nominal phrase. Under the traditional view of phrase structure (e.g., Bowers, 1993), this would mean that clauses and noun phrases can be assumed to involve the abstract structure in (20), which consists of a predicate phrase PredP, a functional layer FP (including specific projections hosting TMA and modifiers), and an LP (including specific projections hosting discourse particles e.g., focus, topic, cf. Aboh, 2004).

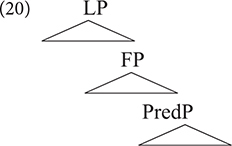

The observations in the previous paragraph suggest that structural linguistic variation is mainly driven by phase properties of LP. The point here is not about a phase parameter, that is, which specific functional head (e.g., D, T, Force) may constitute a phase cross-linguistically. Instead, the relevant point here is that variations within LP expressions point to variations in the internal structure of LP, which may impact FP cross-linguistically. With regard to the clausal domain, such variations seem to correlate with word order patterns, subordination, and possibilities of wh-extractions which have far reaching consequences on the structure of languages. Within the nominal domain, variation within LP can be attributed to licensing of argument DPs cross-linguistically, and how this correlates with bare NP languages versus languages with determiner-NPs, and how these properties relate to other clausal aspects (cf. Bošković, 2008). In the context of our discussion, this amounts to saying that recombination creates more distant structural variants when it comes to the LP. In this regard, a noticeable and well-studied example is Modern English: What makes Modern English a typologically unique West Germanic language is not its FP (i.e., expressions of TMA) but rather the fact that it lost V2: a property of West Germanic LP. It is interesting, however, to note that despite not being a typical V2 language, English does exhibit what Rizzi (1996) refers to as “residual verb second”, that is, the fact that the finite verb (or auxiliary) must occur in second position in certain constructions involving interrogatives or negative inversion (cf. Haegeman, 1995). That English shows such a hybrid property constitutes further evidence in support of the view developed here in terms of recombination and hybrid grammars.

## Conclusion

In this paper, I argue that language acquisition involves contact of idiolects (i.e., contact between individual S-learners leading to contact between different linguistic features in the mind of individual S-learners). Building on Aboh (2015b), I propose that grammars emerge through *recombination*: a fully automated cognitive process which allows S-learners to select linguistic features and recombine them into new syntactic objects as part of their mental hybrid grammars. Immediately observable instances of recombination are illustrated by code-mixing which appears a capacity present in all S-learners. In this regard, I have shown that both neuro-atypical and neuro-typical S-learners exhibit similar production (and arguably processing) patterns, a conclusion already reached by Perecman in the early 80s. What this paper adds to the discussion is the distinction between the role of executive functions as necessary for vocabulary selection, while recombination appears an innate capacity.

Building on this, I further show that while recombination within the TMA domain, traditionally referred to as the INFL-domain, is immune to structural change due to strong Minimality constraints, this does not seem to be the case when it comes to the left periphery, that is, the phase level. I therefore conclude that structural variation of the type that leads to typological variation is a phase-level property. This view accounts for the fact that even though recombination appears “free,” its effects vary depending on the structural domain that it applies to. While the discussion here mainly focuses on syntax, one can imagine similar recombination patterns in semantics and phonology, and how these are constrained cross-linguistically.

The approach developed in this paper makes clear what core aspects of language are common to neuro-typical and neuro-atypical S-learners. There has been a tendency in the literature to study neuro-atypical S-learners only from the perspective of what they “lack” or “fail to exhibit.” By focusing on what is common to both neuro-typical and neuro-atypical S-learners, this paper sheds light on the relation between fundamental aspects of language and peripheral ones, that is, what is core and undamageable versus what is peripheral (and presumably damageable and variable).

The discussion in this paper mainly focuses on mixing patterns found in certain aphasic patients. A more comprehensive work is needed to establish a typology of the different neuro-atypical cognitive phenotypes, and in conjunction with this, a typology of their mixing patterns. Such a typology is necessary to establish the degrees to which neuro-typical and neuro-atypical cognitive phenotypes exhibit (dis)similar recombination patterns.

Another important question that arises under the theory of clause structure and recombination presented here, and which merits further investigation, is how the different domains identified in (20) are processed. Recent studies suggest that syntactic processing involves several brain regions which are also involved in other cognitive tasks even though together they may form a tight network specialized in linguistic computation (e.g., Vigliocco, 2000; Kaan and Swaab, 2002; Friederici, 2011). If syntactic processing results from a diffuse network, we may further wonder whether phrase structures, i.e., LP, FP, and PredP are all processed similarly. While this question is not discussed in current Minimalist theories, the view of recombination developed in this paper is compatible with the assumption that LP versus FP/PredP might be processed differently. There is now a body of literature demonstrating a more articulate neurobiology of language that suggest such a possibility, and I hope this paper will generate further discussion on the matter.

## Author ContribuTions

The author confirms being the sole contributor of this work and has approved it for publication.

## Conflict of Interest

The authors declare that the research was conducted in the absence of any commercial or financial relationships that could be construed as a potential conflict of interest.

## Footnotes

1 Interestingly, *Àjàc$´\varepsilon$* is an approximation by Yoruba or (Nago) speakers of the expression *àjá cè* which itself is a Gbe expression meaning *my Adja* (i.e., the original location of the Gbe people). These expressions are indicative of the interaction between the founding populations, and how speakers of different languages approximate the languages of their neighbors.

2 Like *Àjàc$´\varepsilon$*, Porto-Novo is the French rendering of Puerto Nuevo, the name given to the town by the Portuguese, prior to colonization by the French.

3 This seems compatible with how S-learners feel about code-switching/mixing. When asked about this mode, speakers report finding it “easy” or indicate that they adopt it out of “laziness,” “exhaustion,” or “excitement” (cf. Dorleijn, 2017 and references cited therein). Accordingly, code-switching/mixing happens when S-learners do not want to or cannot control for language selection entirely.

4 I follow the Minimalist convention in which “lexical item” refers to both content and functional items.

5 H.B., was an 80 years old male patient, born in Cameroon. His parents were German, and he spoke German natively, then French as L2, and English as L3, when he settled in the US at the age of 18. English was his most active language.

6 It is very well possible that neuro-atypical speakers showing pathological code-mixing produce some other patterns that are structurally different or absent in neuro-typical populations, or that neuro-typical speakers produce patterns that are never found in neuro-atypical speakers. I’m not, however, aware of any study that discusses such structural dissimilarities systematically.

7 It is not clear in the literature I’ve consulted whether such mixing occurs at the morphemic level (e.g., a root and its affix being pronounced by the intonation of two different languages).

8 In terms of this paper, this capacity is subsumed by recombination.

9 I thank a reviewer of *Frontiers* for her/his comments on this formulation.

10 Outputs of recombination in apparent “monolinguals” can also be illustrated by speech errors (cf. Pfau, 2009 and references therein).

11 In order to keep the discussion manageable, I leave aside potential cases of recombination in which lexical components co-vary (e.g., phonology vs. morphosyntax or morphosyntax vs. semantics). I hope to return to these cases in future work.

12 The interested reader can consult the Yale Grammatical Diversity Project: English in North America, https://ygdp.yale.edu/for more documented variants.

13 A reviewer correctly remarks that such “ungrammatical” recombination may well be observed in some neuro-atypical speakers, e.g., in patients with Huntington’s disease (cf. Németha et al., 2012). I thank this reviewer for bringing this to my attention and I hope that future work will shed better light on this question.
